# Transcriptome analysis reveals regional and temporal differences in mucosal immune system development in the small intestine of neonatal calves

**DOI:** 10.1186/s12864-016-2957-y

**Published:** 2016-08-11

**Authors:** Guanxiang Liang, Nilusha Malmuthuge, Hua Bao, Paul Stothard, Philip J. Griebel, Le Luo Guan

**Affiliations:** 1 Department of Agricultural, Food and Nutritional Science, University of Alberta, Edmonton, AB T6G 2P5 Canada; 2 Vaccine and Infectious Disease Organization, University of Saskatchewan, Saskatoon, SK Canada; 3 School of Public Health, University of Saskatchewan, Saskatoon, SK Canada

**Keywords:** Neonatal calves, Gene expression, miRNA, Mucosal immune system, RNA-Seq

## Abstract

**Background:**

Postnatal development of the mammalian mucosal immune system is crucial for responding to the rapid colonization by commensal bacteria and possible exposure to pathogens. This study analyzed expression patterns for mRNAs and their relationship with microRNAs (miRNAs) in the bovine small intestine during the critical neonatal period (0 to 42 days). This analysis revealed molecular mechanisms regulating the postnatal development of the intestinal mucosal immune system.

**Results:**

Small intestine samples (jejunum and ileum) were collected from newborn male, Holstein calves immediately post-partum (*n* = 3) and at 7 (*n* = 5), 21 (*n* = 5), and 42 (*n* = 5) days of age and the transcriptomes were profiled using RNA-Seq. When analyzing all time points collectively, greater expression of genes encoding the complement functional pathway, as well as lower expression of genes encoding Toll-like receptors and NOD-like receptors were observed in the jejunum when compared to the ileum. In addition, significant changes in the expression of immune-related genes were detected within the first week post-partum in both jejunum and ileum. For example, increased expression of genes encoding tight junction proteins (claudin 1, claudin 4 and occludin), an antimicrobial peptide (Regenerating Islet-Derived 3-γ), NOD-like receptors (NACHT, LRR and PYD domain-containing protein 3), regulatory T cell marker (forkhead box P3), and both anti-inflammatory (interleukin 10) and pro-inflammatory (interleukin 8) cytokines was observed throughout the small intestine of 7-day-old calves when compared to newborn calves. Moreover, the expression of mucosal immune-related genes were either positively or negatively correlated with total bacterial population depending on both intestinal region and age. The integrated analysis of miRNAs and mRNAs supported the conclusion that miRNAs may regulate temporal changes in the expression of genes encoding tight junction proteins (miR-335), cytokines (miR-335) and bacterial recognition (miR-100) during the first week of small intestine development.

**Conclusion:**

The rapid development of transcriptional differences between jejunum and ileum reveal that these two intestinal regions make distinct contributions to the intestinal mucosal immune system during the early neonatal period. In addition, transcriptome analysis indicates that the first week after birth is a very dynamic developmental period for the intestinal mucosal immune system and these changes may be regulated by both miRNAs and microbial colonization. Findings from this study indicate that a detailed analysis of both the abundance and diversity of the colonizing microbiome may be necessary to understand factors regulating the rapid development of the mucosal immune system during the first week of life.

**Electronic supplementary material:**

The online version of this article (doi:10.1186/s12864-016-2957-y) contains supplementary material, which is available to authorized users.

## Background

Newborn calves are susceptible to a variety of bacterial and viral enteric infections, due to an immature immune system and a possible failure in the passive transfer of maternal antibodies [1]. The mucosal immune system in the bovine small intestine consists of both organized lymphoid tissues, such as Peyer’s patches (PPs), and diffuse lymphoid tissue, which distributes throughout the lamina propria and intraepithelial compartments [2]. The mucosal immune system is the first line of defence, protecting the host from enteric infection and the commensal microbiome by providing physical barriers and activating both innate and adaptive immune responses [3]. The neonatal intestinal mucosal immune system undergoes rapid change in response to microbial colonization, exposure to potential pathogens, and environmental factors including toxins and dietary components, such as colostrum [4]. A major challenge for the developing mucosal immune system is discriminating between commensal microbes and pathogenic microbes to ensure protection of the host [4], without disrupting vital intestinal functions required for maintaining health. However, little is known about the mucosal immune function during early postnatal developmental period in ruminants.

Intestinal mucosal immune system development has been studied in mice and humans [5]. When compared to mice, the ruminant intestinal mucosal immune system displays several unique developmental features that are shared by a variety of other mammalian species, such as humans. Two developmentally and structurally distinct types of PPs are located in the jejunum and terminal small intestine [6]. Furthermore, PP development begins in utero, in the absence of the commensal microbiome, with organized lymphoid follicles present at the time of birth. In ruminants, the continuous PPs, located in the ileum, function primarily as a site for generation of the pre-immune B cell, while the discreet PPs, distributed throughout the jejunum, function as induction sites for the generation of IgA plasma cells [7]. It remains unknown to what extent these regional differences in organized mucosa-associated lymphoid tissues influence host responses to the commensal microbiome or how the commensal microbiome regulates post-natal development of PP function. The ileum has, however, been identified as a preferential site of neonatal infection by a variety of pathogens, including *Salmonella* spp. [8] and *Mycobacterium avium* subsp. *Paratuberculosis* infection [9].

Significant age-dependent changes in the number and distribution of mucosal leukocyte sub-populations was reported when comparing cells isolated from the small intestine of 3 to 5-week-old versus 6 month old calves [10]. Furthermore, an age-dependent down-regulation of mRNA expression for several Toll-like receptors and antimicrobial peptides was observed when comparing intestinal tissues collected from 3-week old versus 6-month old calves [11]. In addition, our recent study described significant changes in bovine intestinal microRNA (miRNA) expression throughout the post-natal period (0 to 6 weeks) and these studies suggested a possible involvement of miRNAs in regulating mucosal immune system development [12]. However, the molecular mechanisms regulating both regional and temporal differences in the early post-natal development of the bovine intestinal mucosal immune system has not been investigated.

Identifying regulatory relationships between miRNAs and their corresponding mRNA targets is critical for understanding developmental processes that occur throughout the small intestine in neonatal calves. Therefore, in this study we investigated regional and temporal changes in the intestinal function of newborn calves by analyzing global changes in gene expression (transcriptome) in both jejunum and ileum from birth until 42 days postpartum. This analysis focused specifically on genes related to the mucosal immune system and included an integrated analysis of both of miRNAs and mRNAs expression. This integrated analysis provided an opportunity to explore the potential regulatory role of miRNAs in this developmental process.

## Results

### Small intestine transcriptomes

**Fig. 1:**
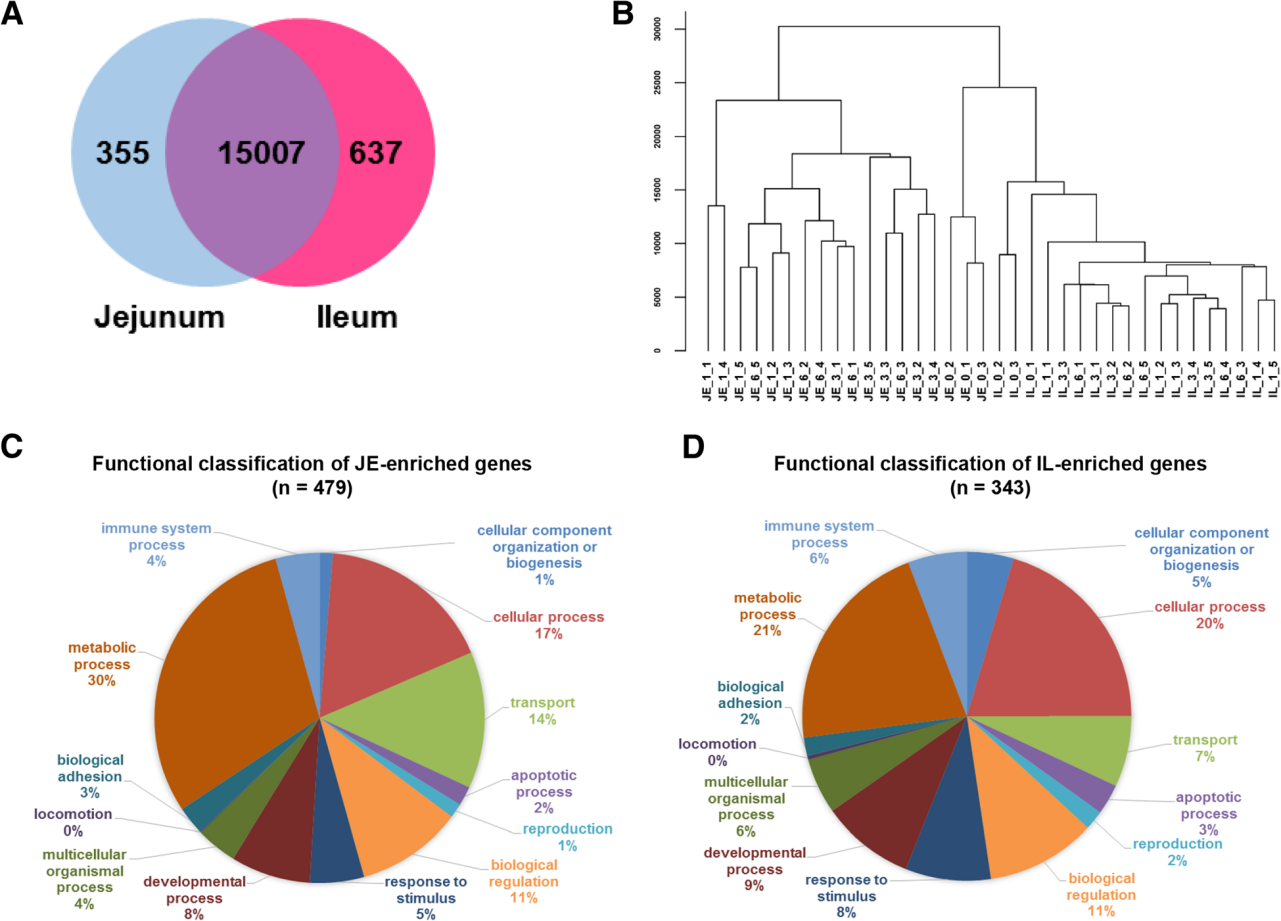
Regional difference of the transcriptome profiles between the jejunum (JE) and ileum (IL). **a** Venn diagram of expressed genes. **b** Hierarchical cluster of mRNA expression. Each ID represents one animal. “JE_0_1” means the jejunum sample from D0 animal No.1 (D0). **c** Functional classification of JE-enriched genes. **d** Functional classification of IL-enriched genes

A total of 1,350 million high-quality 100-bp paired-end reads were obtained from 36 libraries (jejunum and ileum samples from Holstein bull calves at birth (D0; *n* = 3), 7 days post-partum (D7; *n* = 5), 21 days post-partum (D21; *n* = 5) and 42 days post-partum (D42; *n* = 5)), with an average of 38.2 ± 8.3 million reads per library. Of all the reads, ~81.5 % from the jejunum and ~82.5 % from the ileum, were mapped to the bovine genome (UMD 3.1). Based on the normalized data, expression of 15,362 and 15,644 genes were detected (with CPM (counts per million mapped reads) > 1 in at least 50 % of samples) in the jejunum and ileum, respectively. Among these genes, 15,007 genes were commonly detected in both jejunum and ileum (Fig. 1a). The most relevant gene ontology (GO) terms of top 3000 highly expressed genes were “metabolic process” and “protein transport” (Additional file 1).

### Regional and temporal differential expression of genes

The generated transcriptome profiles revealed a clear clustering pattern depending on the small intestinal region regardless of calf developmental stages (Fig. 1b). When the regional difference was further explored within each age category using edgeR [13] (fold change > 2, false discovery rate (FDR) < 0.05), a total of 479 genes showed higher expression in jejunum than ileum (JE-enriched, Fig. 1c), whereas 343 genes had higher expression in ileum than jejunum (IL-enriched, Fig. 1d) at one or more of the time points (Additional file 2). Functional classification showed that 30 % and 14 % of the JE-enriched genes were related to “metabolic process” and “transport,” respectively (Fig. 1c), while fewer IL-enriched genes (21 % and 7 %) were related to these two functions (Fig. 1d). Further, 4 % of JE-enriched and 6 % of IL-enriched genes were related to “immune system process”.

**Fig. 2:**
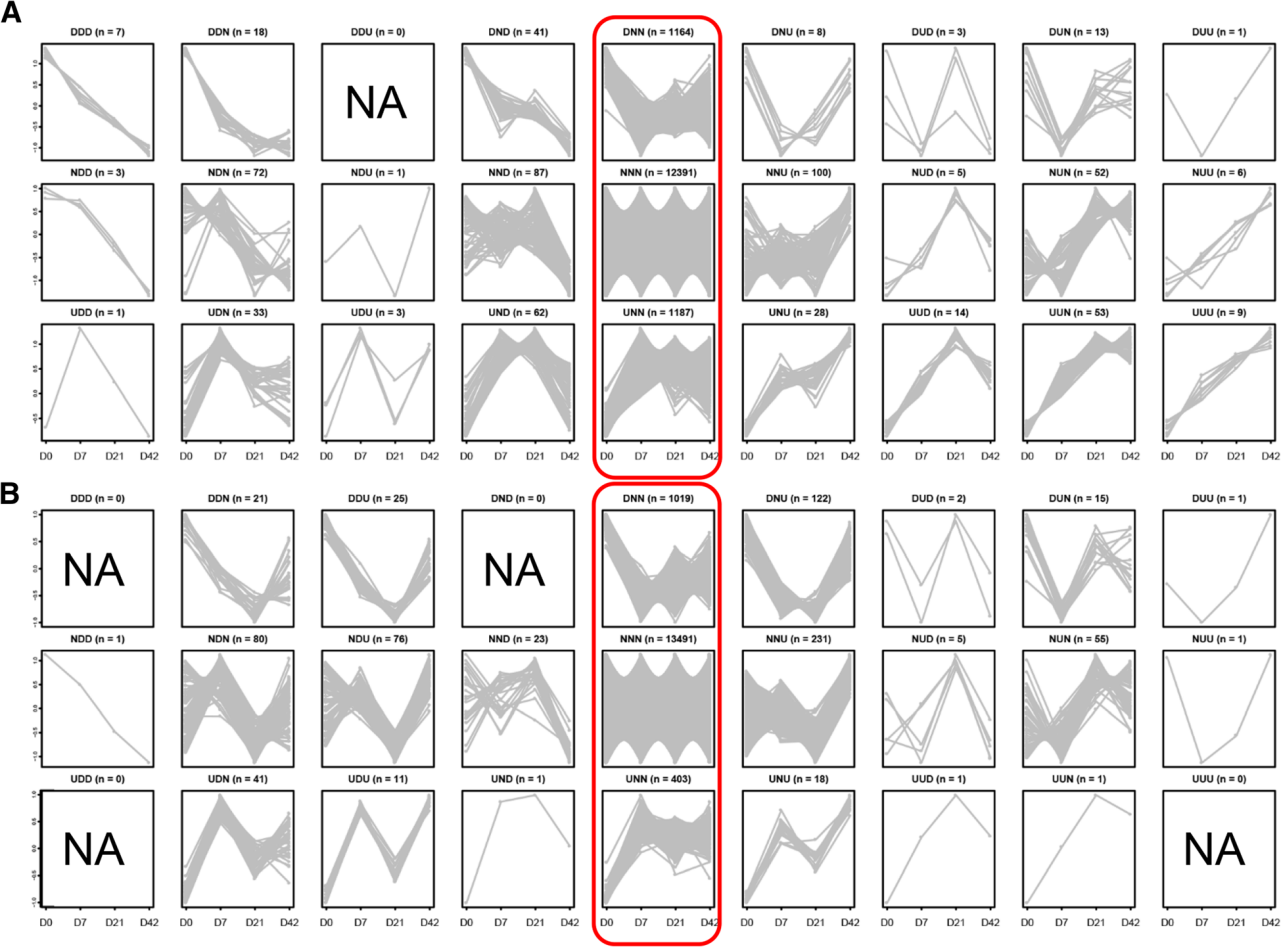
Temporally DE genes expression patterns for jejunum **a** and ileum **b**. All the expressed genes were categorized into 27 expression patterns based on the temporally DE analysis (fold change > 2 and FDR < 0.05). “U” means the genes were DE and upregulated; “N” means the genes were not DE; and “D” means the genes were DE and dwonregulated. The order of each pattern follows the comparison between D7 vs D0, D21 vs D7, and D42 vs D42. X-axis depicts four ages and Y-axis depicts the fold change of each gene. “NA” in the square means no genes were categorized into that pattern

In addition to the regional variations observed, the transcriptome profiles displayed rapid temporal changes within both small intestinal regions during the first six weeks of life (Additional file 3). These temporal changes were investigated by sequentially comparing the expression of genes between proximal age groups (D7 *vs* D0; D21 *vs* D7; D42 *vs* D21). The expression patterns of jejunal differentially expressed (DE) genes were categorized into 26 patterns (Fig. 2a), while 23 patterns were observed for that of ileal DE genes (Fig. 2b) with “U”, “D” and “N” representing the genes upregulated, downregulated, and not differentially expressed, respectively (significant differences were declared at fold change > 2, FDR < 0.05). The order of the combination represents the expression pattern change following the comparisons D7 *vs* D0, D21 *vs* D7 and D42 *vs* D21. For example, genes that were categorized into expression pattern “UND” means that these genes were upregulated in D7 *vs* D0, unchanged in D21 *vs* D7, and downregulated in D42 *vs* D21. From all the patterns observed, three patterns represented 96 % of genes for both tissues. The largest numbers of genes, representing 80 % of total expressed genes in the jejunum (Fig. 2a) and 86 % of total expressed genes in the ileum (Fig. 2b), displayed no change in expression (pattern “NNN”) during the first six weeks. The next largest groups of genes belonged to patterns “UNN” and “DNN” and displayed changed expression only during the first week of life. GO terms enrichment revealed that genes that belonged to patter “UNN” were mainly related to “immune system process” in the jejunum and ileum (Additional file 4). And genes that belonged to pattern “DNN” were mainly related to “developmental process” in the jejunum and ileum (Additional file 4).

### Systematic analysis of regional and temporal differences in immune-related gene expression

**Fig. 3:**
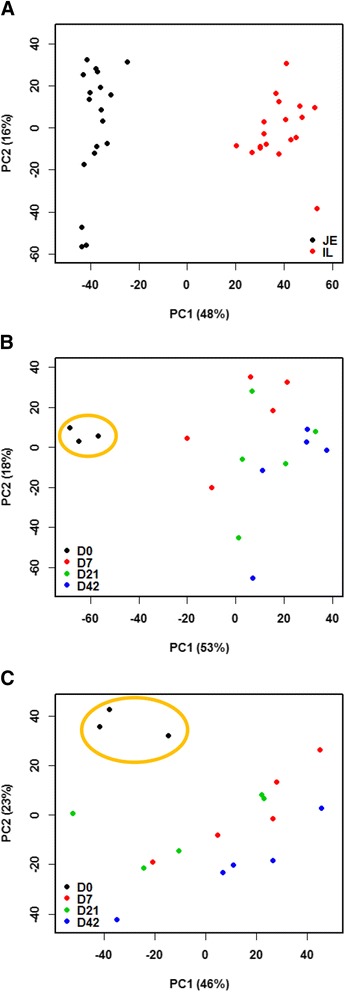
PCA plot of all the immune-related genes. The X and Y-axis represent the first two principle components. The percentage value in the bracket represents the percentage of variance explained by that principle component. **a** PCA plot of immune-related genes for the jejunum (black dots) and ileum (red dots). **b** PCA plot of immune-related genes for the jejunum at different ages. **c** PCA plot of immune-related genes for the ileum at different ages

To further understand regional and temporal differences in mucosal immune system development in the small intestine of newborn calves, a total of 3314 and 3306 expressed immune-related genes (CPM > 1 in at least 50 % samples) in the jejunum and ileum, respectively, were subjected to further analysis (Additional file 5). Similar to the whole transcriptome profiles (Fig. 1b), the expression of immune-related genes also displayed a clear separation between jejunum and ileum based on PCA analysis (Fig. 3a). When expression of these genes was compared between the two regions, 214 genes (105 JE-enriched and 109 IL-enriched) were identified as regionally DE immune-related genes. The KEGG pathway analysis showed that the JE-enriched immune-related genes were related primarily to “complement and coagulation cascades” (Additional file 6), whereas the IL-enriched immune-related genes were mainly relevant to “B cell receptor signalling pathway” (Additional file 6).

The immune-related genes identified above within each small intestinal region were then compared among the four ages to further determine if there was a temporal expression pattern. The expression of immune-related genes at birth was different from the three other developmental stages in both jejunum (Fig. 3b) and ileum (Fig. 3c) as highlighted by the orange circle. Further analysis of the expression patterns of temporally DE immune-related genes showed that 867 were DE and 2,431 were unchanged in the jejunum, while 605 were DE and 2689 were unchanged in the ileum during three postnatal periods (D7 *vs* D0, D21 *vs* D7 and D42 *vs* D21). The KEGG pathway analysis showed that temporally DE immune-related genes in the jejunum were mainly related to “complement and coagulation cascades” (Additional file 7), while the temporally DE immune-related genes in the ileum were mainly related to “ECM-receptor interaction” (Additional file 7).

### Expression changes in genes encoding tight junction proteins, antimicrobial peptides and mucins

**Fig. 4:**
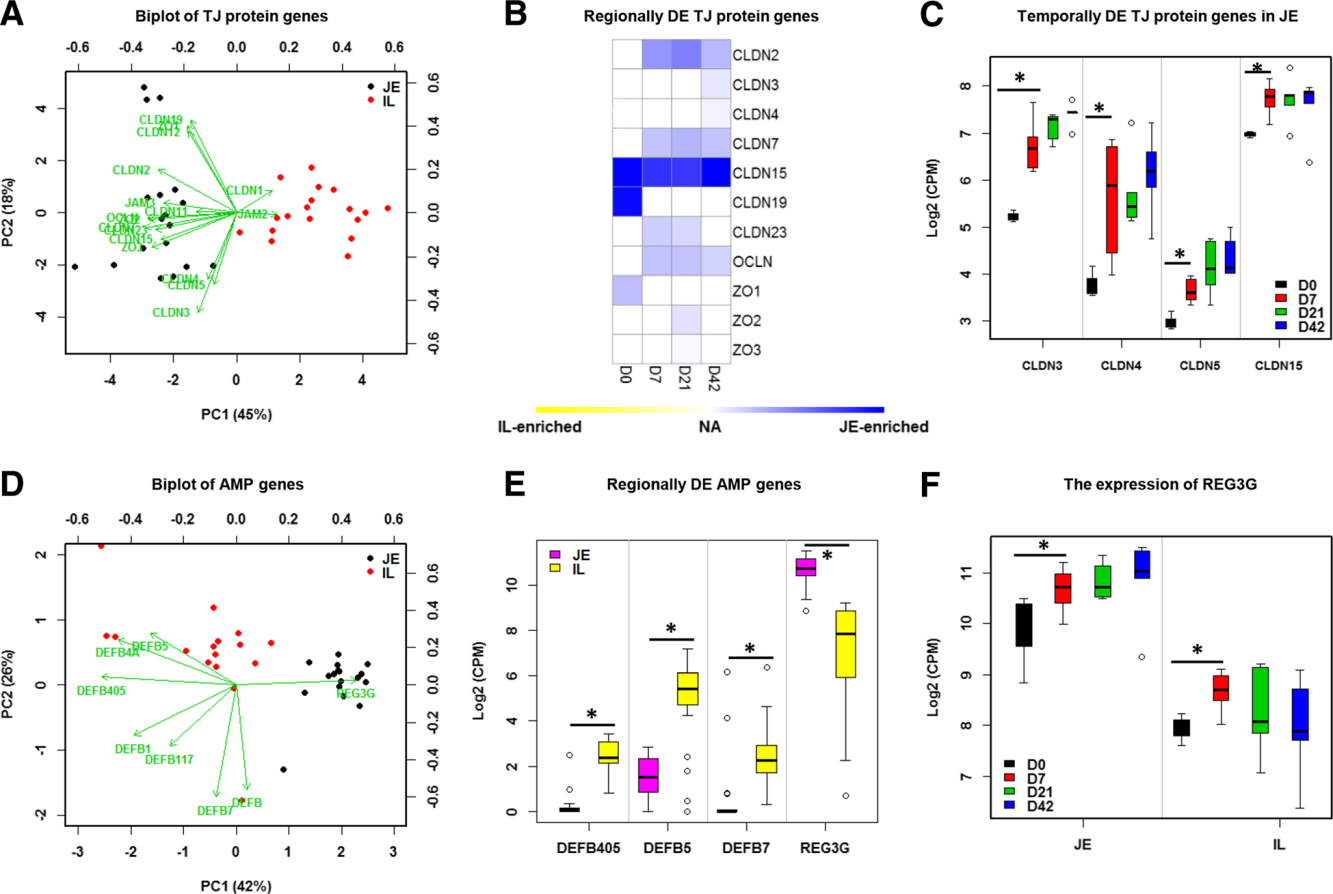
The expression pattern TJ protein genes. **a** Biplot of TJ protein genes. The X and Y-axis represent the first two principle components. The green arrow points to the samples with higher expression of the gene. **b** Regionally DE analysis of TJ protein genes. Blue means highly expressed in the jejunum and yellow means highly expressed in the ileum. **c** Temporally DE analysis of TJ protein genes. Y-axis depicts the gene expression level (log2 (CPM)). “*” means significantly difference identified by temporally DE analysis. **d** Biplot of AMP genes. **e** Regionally DE analysis of AMP genes. “*” means significantly difference identified by regionally DE analysis. **f** The expression pattern of REG3G in both jejunum and ileum. “*” means significantly difference identified by temporally DE analysis

Expression of 16 genes encoding tight junction (TJ) proteins, including the claudin family (*CLDN1*, *CLDN2*, *CLDN3*, *CLDN4*, *CLDN5*, *CLDN7*, *CLDN11*, *CLDN12*, *CLDN15*, and *CLDN23*), occludin (*OCLN*), zonula occluden family (*ZO1*, *ZO2*, and *ZO3*) and the junctional adhesion molecule family (*JAM2* and *JAM3*) were detected in jejunum and ileum. Expression of TJ protein genes segregated by small intestinal region, due to higher expression levels in the jejunum compared to the ileum (Fig. 4a). DE analysis of TJ protein genes also revealed that *CLDN2*, *CLDN3*, *CLDN4*, *CLDN7*, *CLDN15*, *CLDN19*, *CLDN23*, *OCLN*, *ZO1*, *ZO2* and *ZO3* were JE-enriched genes, while no IL-enriched genes were identified (Fig. 4b). Among these DE genes, *CLDN15* was highly expressed in jejunum versus ileum at all ages examined (Fig. 4b). In addition, the expression of *CLDN3*, *CLDN4*, *CLDN5* and *CLDN15* was upregulated at D7 when compared to D0 in the jejunum but no further changes after D7 were detected (Fig. 4c). In contrast, none of these genes were temporally DE in the ileum.

For genes encoding antimicrobial peptides (AMPs), the expression of β-defensins (*DEFB*, including *DEFB1*, *DEFB4A*, *DEFB5*, *DEFB7*, *DEFB117*, *DEFB405*) and regenerating islet-derived 3-γ (*REG3G*) were detected by RNA-Seq (Fig. 4d). Between the two intestinal regions, *DEFB405*, *DEFB5* and *DEFB7* were IL-enriched genes, while *REG3G* was a JE-enriched gene (Fig. 4e). Besides, *REG3G* was upregulated from D0 to D7 but expression did not increase further after D7 in either the jejunum or ileum (Fig. 4f). None of the *DEFB* genes were identified as temporally DE.

Genes that encode mucins, such as *MUC1*, *MUC3A*, *MUC4*, *MUC12*, *MUC13*, *MUC19*, *MUC20* and *MUC21*, were detected by RNA-Seq (CPM > 1 in at leaset 50 % samples). No regionally DE mucin genes were identified. *MUC12* showed 3.6-fold decreased in jejunum and *MUC13* showed 2.3-fold decrease in ileum within the first week after birth.

### Toll-like receptor and NOD-like receptor genes expression pattern

**Fig. 5:**
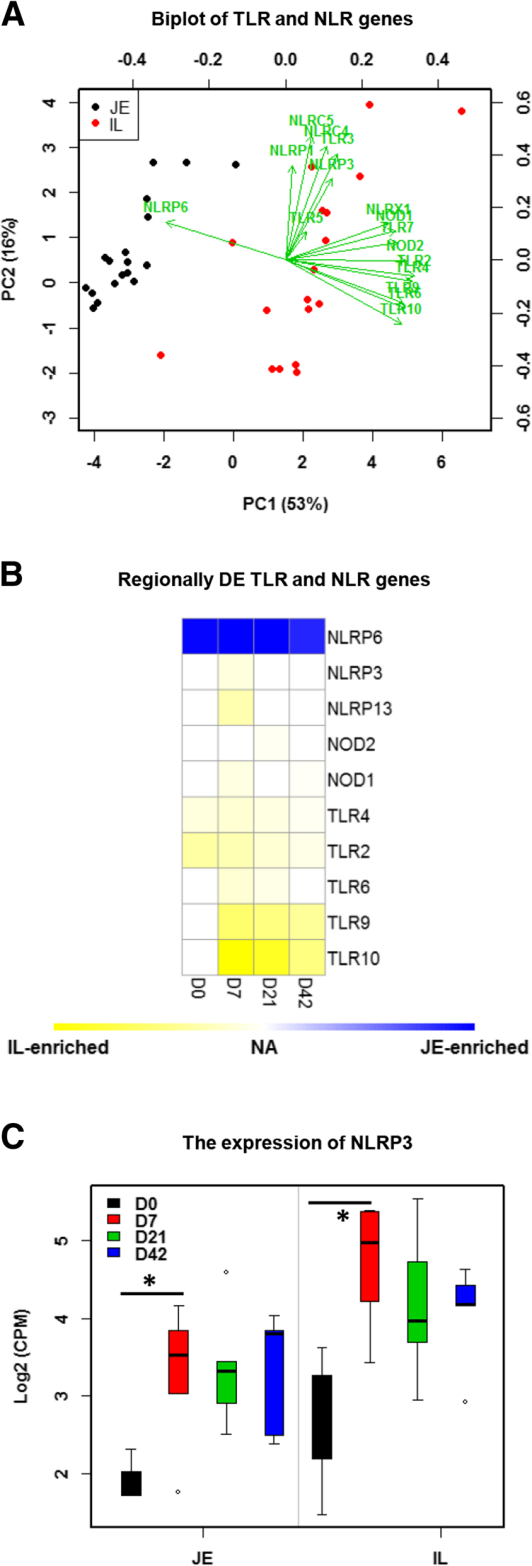
The expression pattern TLR and NLR genes. **a** Biplot of TLR and NLR genes. The X and Y-axis represent the first two principle components. The green arrow points to the samples with higher expression of the gene. **b** Regionally DE analysis of TLR and NLR genes. Blue means highly expressed in the jejunum and yellow means highly expressed in the ileum. **c** Boxplot of expression pattern of NLRP3 in the jejunum and ileum. Y-axis depicts the gene expression level (log2 (CPM)). “*” means significantly difference identified by temporally DE analysis

Expression of Toll-like receptor (TLR) family genes including *TLR2*, *TLR3*, *TLR4*, *TLR5*, *TLR6*, *TLR7*, *TLR9*, and *TLR10*, and NOD-like receptor (NLR) family genes including *NOD1*, *NOD2*, *NLRC4*, *NLRC5*, *NLRP1*, *NLRP3*, *NLRP6*, and *NLRX1* were detected by RNA-Seq (Fig. 5a). When comparing the two intestinal regions, *NLRP6* was a JE-enriched gene, while *TLR2*, *TLR4*, *TLR6*, *TLR9*, *TLR10*, *NLRP3*, *NLRP13*, *NOD1* and *NOD2* were IL-enriched but this difference varied with age (Fig. 5b). When gene expression was compared for different ages, *NLRP3* was temporally DE in both jejunum and ileum and was significantly higher at D7 versus D0 with no further change in expression after D7 (Fig. 5c). None of the TLR genes were identified as temporally DE genes.

### Expression patterns for T and B cell lineage-specific genes and IgA production-related genes

**Fig. 6:**
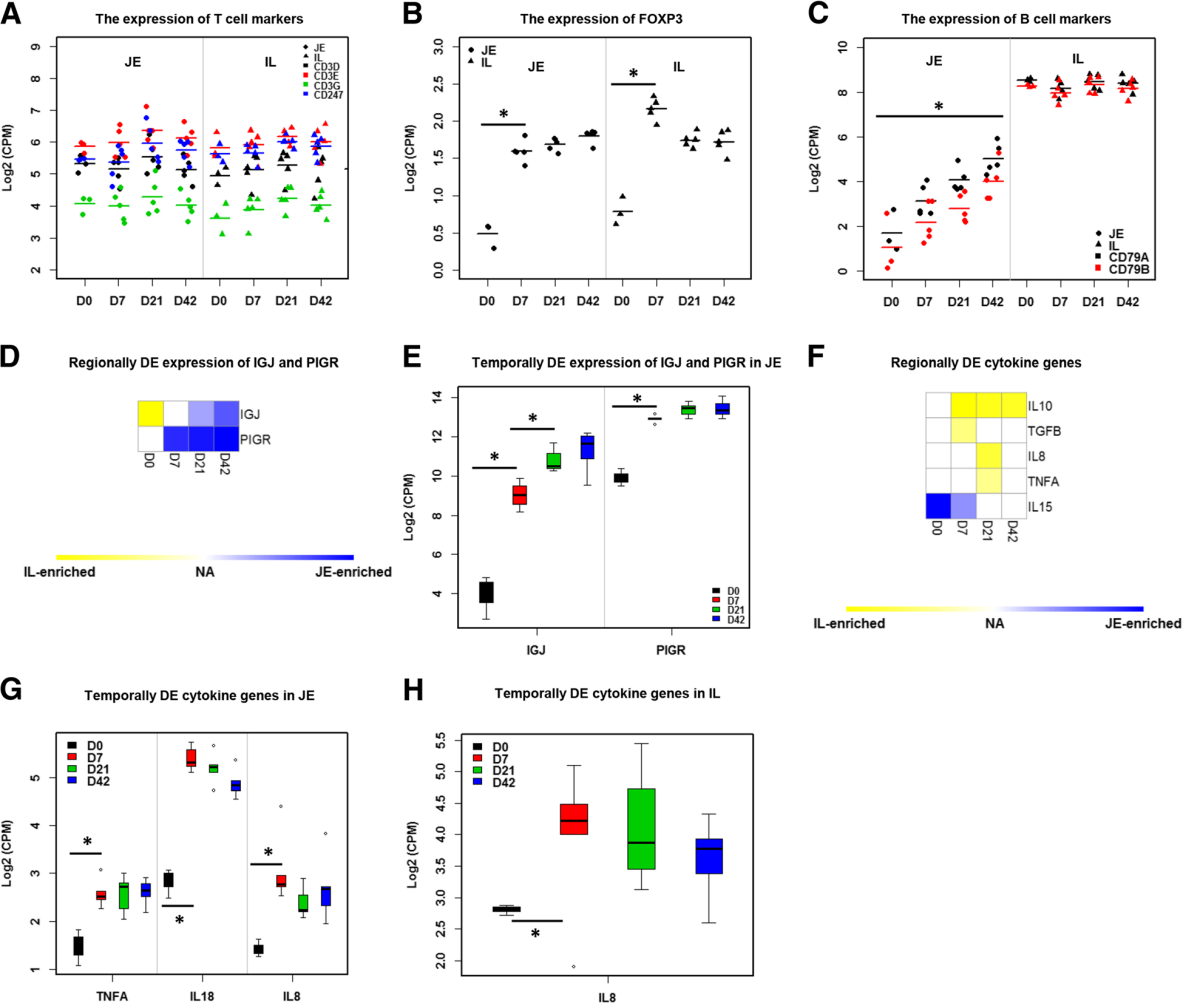
The expression pattern of adaptive immune-related genes and cytokine genes. **a** Dot plot of the expression of T cell markers (CD3 subunits and CD247). Each dot represents the gene expression each sample. Y-axis represents the gene expression level (log2 (CPM)). Y-axis represents the ages. **b** Dot plot of the expression of Treg marker (FOXP3). “*” means significantly difference identified by temporally DE analysis. **c** Dot plot of the expression of B cell markers (CD79 subunits). **d** Regionally DE analysis of IGJ and PIGR. Blue means highly expressed in the jejunum and yellow means highly expressed in the ileum. **e** Temporally DE analysis of IGJ and PIGR in the jejunum. **f** Regionally DE analysis of cytokine genes. **g** Temporally DE analysis of cytokine genes in the jejunum. **h** Temporally DE analysis of cytokine genes in the ileum

Among the T cell lineage-specific genes detected, no temporal or regional effect was observed for the expression of Cluster of Differentiation (*CD*) *3D*, *CD3E*, *CD3G* or *CD247* (Fig. 6a). A significant increase in forkhead box P3 (*FOXP3*) gene expression was detected in both jejunum and ileum during the first week of life (Fig. 6b). For the expression of detected B cell lineage-specific genes, *CD79A* and *CD79B* were IL-enriched genes (Fig. 6c). The expression of these genes increased significantly with age in the jejunum (Fig. 6c), and no temporal change was observed for ileum (Fig. 6c).

For genes encoding immunoglobulins, the expression of immunoglobulin J chain (*IGJ*) and polymeric immunoglobulin receptor (*PIGR*) were detected by RNA-Seq in both jejunum and ileum. *IGJ* showed higher expression in the ileum at D0 compared to the jejunum, but subsequently higher expression was detected in the jejunum after D21 (Fig. 6d). Moreover, *PIGR* was a JE-enriched gene from D7 onward (Fig. 6d). Both *IGJ* and *PIGR* showed remarkably increased expression when comparing D7 to D0, and the expression of *IGJ* continued to increase until D21 in the jejunum (Fig. 6e) but neither gene displayed temporal DE in ileum.

### Altered expression patterns for cytokine genes

The expression of interleukin (*IL*)-10,−7,−8,−15, and−18, tumor necrosis factor α (*TNFA*) and transforming growth factor β (*TGFB*) were detected by RNA-Seq in the small intestine samples. *IL8*, *IL10*, *TGFB* and *TNFA* were identified as IL-enriched genes, whereas, *IL15* was a JE-enriched gene (Fig. 6f). The expression of *IL8*, *IL18* and *TNFA* increased within the first week in the jejunum but no further increase was detected after D7 (Fig. 6g). Similarly, the expression of *IL8* increased within the first week but no further changes were detected in ileum collected from older animals (Fig. 6h).

### Integrated analysis of miRNA and mRNA expression

In our previous study, the majority of temporally DE miRNAs were identified in D7 *vs* D0 comparison when analyzing the same tissue samples used for the present study [12]. In the present study, we focused on the potential regulatory role of miRNAs during the first week due to the dynamic changes in gene expression observed during this same time interval. Possible regulatory relationships between DE miRNAs (Additional file 8, obtained from our previously published paper [12]) and DE mRNAs (Additional file 3) were identified based on two criteria: computational target prediction and DE miRNAs displaying opposite expression patterns from their predicted targets. In total, 3206 miRNA-mRNA (83 miRNAs and 1180 mRNAs) pairs and 1367 miRNA-mRNA (73 miRNAs and 570 mRNAs) pairs were identified in the jejunum and ileum, respectively. The miRTarBase was used to further identify the miRNA-mRNA regulatory pairs that have been experimentally validated by previous studies [14]. In total, 36 miRNA-mRNA (10 miRNAs and 36 mRNAs) pairs and 10 miRNA-mRNA (4 miRNAs and 10 mRNAs) pairs were identified in the jejunum and ileum, respectively (Additional file 9). *CLDN1*, *CLDN4*, *IL8* (the targets of miR-335) and *NLRP3* (the target of miR-100) were related to the barrier and mucosal immune functions (Additional file 9). When their expression was compared, the expression of miR-335 was downregulated (CPM from 66.8 ± 11.9 to 22.3 ± 8.9), and its predicted targets, *CLDN1* (CPM from 2.1 ± 0.8 to 6.1 ± 1.3) and *CLDN4* (CPM from 44.2 ± 1.9 to 77.2 ± 12.6), showed upregulation in the ileum within the first week post-partum. Meanwhile, miR-335 was also predicted to target *IL8* (CPM from 6.0 ± 0.2 to 18.0 ± 4.9), which was upregulated in the ileum within the first week. In addition, miR-100 (CPM from 1600.8 ± 61.1 to 745.9 ± 57.0) was downregulated while its predicted target gene, *NLRP3* (CPM from 6.5 ± 2.3 to 27.2 ± 7.5), was upregulated in the jejunum.

### Validation of differentially expressed mRNAs

**Fig. 7:**
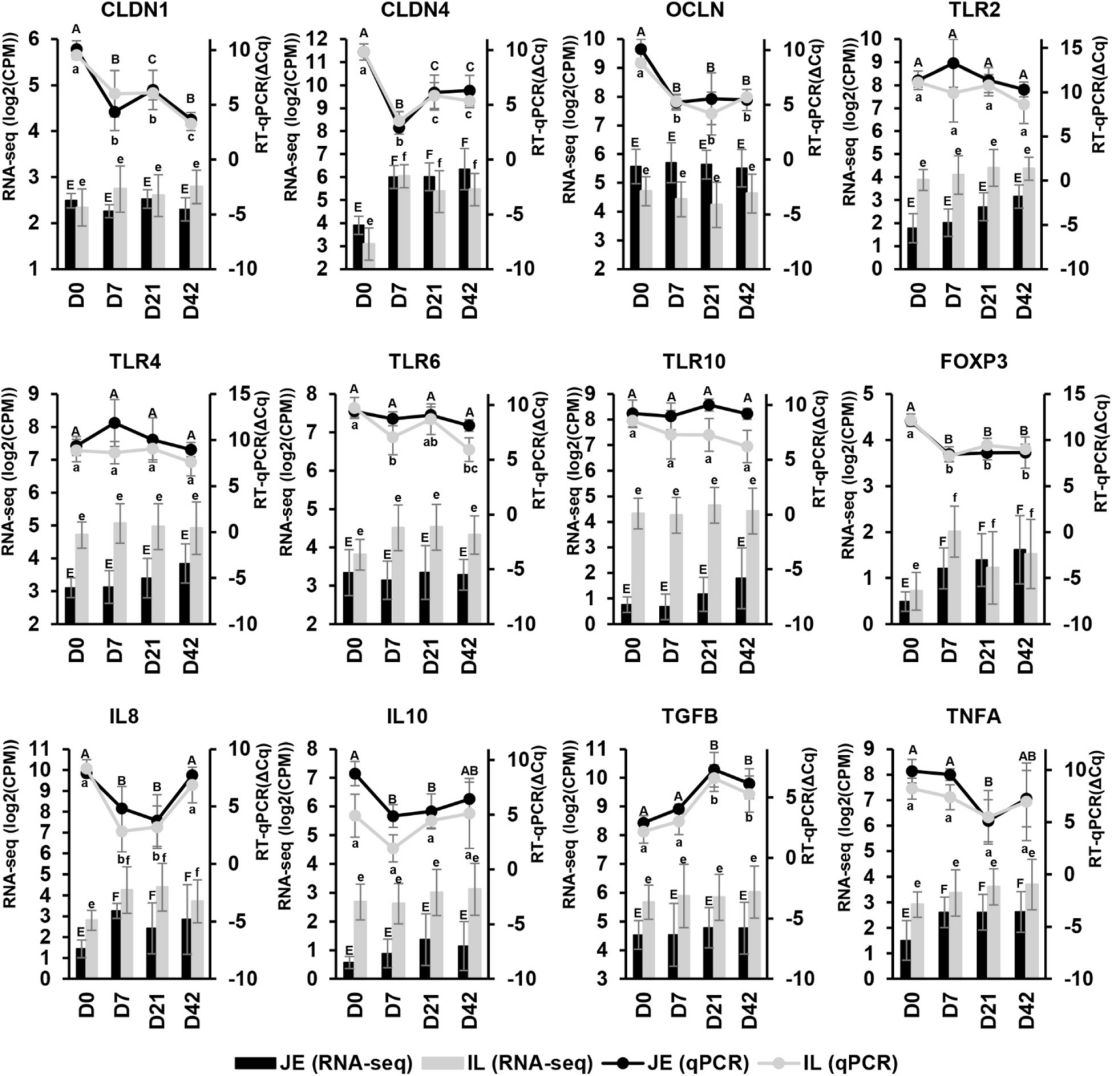
RT-qPCR validation of selected DE genes identified by RNA-Seq. The gene expressions detected by RT-qPCR are shown by line graphs (*black*: jejunum; *grey*: ileum) on the top and values are shown on the right Y-axis as relative expression (ΔCq). Lower ΔCq values represent higher gene expression levels and vice versa. The gene expressions detected by RNA-Seq are shown by bar graphs (*black*: jejunum; *grey*: ileum) on the bottom and values are shown on the left Y-axis as log2 (CPM). A, B, C - indicate significant expression difference detected via RT-qPCR between different ages in the jejunum. a, b, c - indicate significant expression difference detected via RT-qPCR between different ages in the ileum. E, F - indicate significant expression difference detected via RNA-Seq between different ages in the jejunum. e, f - indicate significant expression difference detected via RNA-Seq between different ages in the ileum. Data are presented as Mean ± Standard deviation. Significantly regional difference were not labelled in the figure. TLR2, TLR4, TLR6, TLR10 and IL10 were validated as IL-enriched genes by RT-qPCR (ANOVA, *P* < 0.05)

Reverse transcription quantitative real-time PCR (RT-qPCR) analysis was used to validate the expression of the following genes: *CLDN1* and *CLDN4* from TJ proteins; *TLR2*, *TLR4*, *TLR6* and *TLR10* from TLR gene family; *FOXP3* as Treg marker; *IL8*, *IL10*, *TGFB*, *TNFA* for cytokines. Expression of *TLR2*, *TLR4*, *TLR6*, *TLR10* and *IL10* was significantly higher (*P* < 0.05) in the ileum than the jejunum, which was consistent with RNA-Seq data (Fig. 7). Although *CLDN4* and *OCLN* were identified as JE-enriched genes by RNA-Seq, no significant regional difference was detected by RT-qPCR (Fig. 7). The RNA-Seq showed that the expression of *CLDN4*, *FOXP3* and *IL8* increased from D0 to D7 in both jejunum and ileum, and this observation was validated by RT-qPCR (Fig. 7). In addition, RT-qPCR results revealed temporally DE genes within the first week that were not detected by RNA-Seq. For example, the expression *CLDN1* and *OCLN* were up-regulated within the first week post-partum for both jejunum and ileum. *IL10* and *TLR6* also showed increased expression in the jejunum and ileum, respectively, from D0 to D7 (Fig. 7). Moreover, RT-qPCR also detected temporally DE genes after the first week post-partum which were not identified as significant changes by RNA-Seq. For example, expression of *CLDN1* and *CLDN4* was downregulated when comparing D21 to D7; while *TGFB* was downregulated from D7 to D21 in both intestinal regions (Fig. 7).

### Associations between intestinal content- and tissue-associated total bacteria populations and host gene expression

To test the relationship between host gene expression and bacterial colonization during early life, a Pearson’s correlation analysis was performed between 12 genes validated by RT-qPCR and the 16S rRNA gene copy number for content-associated and tissue-associated total bacteria in the small intestine (Table 1). The content-associated total bacterial population was negatively correlated with *TLR6* and *TNFA* at D7 and with *TLR4*, *OCLN* and *IL8* at D42 in the jejunum (Table 1). No significant correlation was observed between content-associated total bacterial population and host gene expression in the ileum (Table 1). The same analysis for the tissue-associated total bacterial population revealed a negative correlation in the jejunum with *TLR4*, *TLR6*, *TLR10* and *OCLN* at D21 and *TLR6* and *TLR10* at D42 (Table 1). A positive correlation between the tissue-associated total bacterial population was observed for *TLR2*, *TLR4*, *CLDN1* and *OCLN* at D7 in the ileum (Table 1).

**Table 1:** Correlation between content-and tissue-associated total bacteria population and host gene expression

	Content associated						Tissue associated					
	JE			IL			JE			IL		
	D7	D21	D42	D7	D21	D42	D7	D21	D42	D7	D21	D42
TLR2	NS	NS	NS	NS	NS	NS	NS	NS	NS	0.87*	NS	NS
TLR4	NS	NS	−0.84*	NS	NS	NS	NS	−0.89*	NS	0.82*	NS	NS
TLR6	−0.85*	NS	NS	NS	NS	NS	NS	−0.94**	−0.87	NS	NS	NS
TLR10	NS	NS	NS	NS	NS	NS	NS	−0.84*	−0.86	NS	NS	NS
CLDN1	NS	NS	NS	NS	NS	NS	NS	NS	NS	0.85*	NS	NS
CLDN4	NS	NS	NS	NS	NS	NS	NS	NS	NS	NS	NS	NS
OCLN	NS	NS	−0.81*	NS	NS	NS	NS	−0.87*	NS	0.83*	NS	NS
IL8	NS	NS	−0.98**	NS	NS	NS	NS	NS	NS	NS	NS	NS
IL10	NS	NS	NS	NS	NS	NS	NS	NS	NS	NS	NS	NS
TGFB	NS	NS	NS	NS	NS	NS	NS	NS	NS	NS	NS	NS
TNFA	−0.84*	NS	NS	NS	NS	NS	NS	NS	NS	NS	NS	NS
FOXP3	NS	NS	NS	NS	NS	NS	NS	NS	NS	NS	NS	NS

*NS* Not significant

*:*P* < 0.05

**:*P* < 0.01

## Discussion

Prior to weaning, the ruminant small intestine plays a vital role in nutrient absorption and immune function that is similar to monogastric animals. In the present study, whole transcriptome analysis was applied to the analysis of global gene expression patterns for two regions (jejunum and ileum) of the small intestine of neonatal calves. Understanding the whole transcriptome is essential for revealing the breadth of molecular events within cells and tissues, and also for understanding complex developmental processes [15]. Our in-depth analysis revealed significant regional and temporal expression patterns in the whole transcriptome of the bovine small intestine. Because a primary objective was to characterize mucosal immune system development during early life, we focused on the enriched immune-related genes. Therefore, the discussion will focus on 1) regional difference between the two intestinal regions and the distinct temporal changes that occur within each region; and 2) temporal changes that were significantly related to microbial colonization and miRNAs regulation.

Significant differences in expression of immune-related and barrier function-related genes were observed between ileum and jejunum at D0. For example, higher expression of *CLDN15* (fold change = 13.3), *CLDN19* (fold change = 13.7), *REG3G* (fold change = 17.1) and *IL15* (fold change = 5.7) were observed in jejunum versus ileum, while higher expression of *TLR2* (fold change = 5.5) and *TLR4* (fold change = 5.8) were observed in ileum versus jejunum. These results suggest that the mucosal immune system of these two intestinal tissues may have significantly different development in utero. Such change may be associated with the variation in microbial colonization after vaginal delivery, further studies on analyzing the differences of microbial community between the jejunum and ileum at D0 will provide more knowledge on this process.

The transcriptomic analysis identified IL-enriched immune-related genes (compared to jejunum) with functions related to “B cell activation”. Ileum is a site where extensive mucosa-associated lymphoid tissue (MALT) develops in utero and this specific MALT functions as site of a pre-immune B cell development in young calves [16]. Thus, the systematic functional analysis of IL-enriched immune-related genes reflects the functional specialization of the ileal MALT. In contrast to the ileum, greater expression of complement functional pathway-related genes was detected in the jejunum. For example, higher expression of complement component 3 (*C3*) (fold change = 2.5) and complement component 5a (*C5a*) (fold change = 4.8) were observed in jejunum versus ileum. *C3* plays a key role in the activation of the complement system, and *C5a* is crucial in the downstream functions of the complement cascade, which can recruit phagocytic cells to the infection sites [17]. The complement system plays a role in the inflammatory response to microorganisms and the recruitment of phagocytic cells [17]. This is one of the earliest innate immune functions to be fully established in mucosal tissues of newborn human infants [18]. Our transcriptional analysis of the complement functional pathway-related genes reveals that there may be significant differences in early innate immune competence of the jejunum versus ileum of newborn calves.

We also observed significant regional difference for the expression of both T and B cell lineage-specific genes. It was not surprising to observe higher expression of the B cell lineage specific *CD79A* and *CD79B* genes in ileum versus jejunum due to the abundance of B cell lymphoid follicles in the ileal submucosa of newborn calves [19]. The humoral adaptive immune response at mucosal surfaces is mediated primarily by IgA antibody [20], which is secreted by IgA-producing plasma cells. Our transcriptomic analysis revealed that the abundance of IgA complex mRNA was higher in jejunum than ileum (*IGJ* fold change = 2.5; *PIGR* with fold change = 3.2), which is consistent with a previous report that the jejunal but not the ileal PPs function as immune induction sites for IgA antibody responses [21]. Furthermore, we observed an age-dependent increase in the expression of B cell lineage-specific genes in the jejunum but not the ileum, which is consistent with the post-natal development of adaptive immune function in the jejunum. No regional differences in IgA concentration were detected when analyzing samples collected from the lumen of ileal and jejunal samples (data not shown). This result is not consistent with transcriptional data which indicates that the jejunum is the site for greater production (IGJ gene) and secretion (PIGR) of IgA than the ileum (Fig. 6d and e).

The RNA-Seq data also revealed greater expression of TJ protein genes, such as *CLDN3*, *CLDN4*, *OCLN*, *ZO1*, *ZO2* and *ZO3*, in the jejunum than that in the ileum. These genes encode proteins that regulate intestinal permeability [22] and increased expression of these genes may lead to reduce intestinal permeability and increase barrier function. These observations would support the conclusion that the jejunum is better protected than the ileum against the invasion of pathogenic bacteria [23]. However, further studies are needed to compare the expression of these genes at both the transcript and protein level to determine if barrier function varies in these two intestinal regions. In addition, transcript abundance for most of the TLRs, including *TLR2*, *TLR4*, *TLR9* and *TLR10*, was higher in the ileum than jejunum. TLR2 and TLR4 recognize components that were contained in the cell wall of bacteria [24], TLR9 recognizes bacterial DNA [24], and TLR10 was reported to recognize viral components [25]. These TLRs could promote downstream activation of cells in response to either invasive pathogens or the commensal microbiome [24]. Further studies are required to determine which mucosal cell populations express these TLR genes before investigating the implications of these observations. B cells are known to express a wide range of TLRs and it has previously been shown that the gut microbiome is required to maintain B cell proliferation in the ileal Peyer’s patch of sheep [26]. Regardless, the current observations support the conclusion that the jejunum and ileum differ significantly in terms of both innate and adaptive immune responses, bacterial sensing, and barrier functions.

In addition, we observed significant differences in temporal changes in the expression of immune-related genes in both jejunum and ileum during the first week post-partum. This rapid change during the first week after birth may be a regulated by several factors, including microbial colonization, ingestion of colostrum, or programmed ontogenetic development. Increased expression of TJ protein genes, including *CLDN1*, *CLDN4* and *OCLN*, was observed in both jejunum and ileum, when comparing D7 versus D0, suggesting that barrier function increased one week postpartum. This is consistent with a previous report for the jejunum in newborn mice [27]. Furthermore, the strong correlation observed between mRNA expression of TJ proteins and total bacterial number at D7 (expression of *OCLN* was positively correlated with ileal tissue-associated total bacterial density) suggests that the expression of the TJ protein genes may be influenced by the level of bacterial colonization.

Increased expression of the molecular pattern recognition receptor (PRR), *REG3G*, was also observed within the first week post-partum for both the jejunum and ileum. REG3G binds peptidoglycan and is bactericidal for Gram‑positive bacteria [28]. Total bacterial density also increased within the first week (data not shown) and was positively correlated with *REG3G* expression. It was reported that the production of REG3G protein requires the microbial colonization of the small intestine in humans [29]. Increased *REG3G* gene expression may lead to higher REG3G within the first week after birth in calves, which is consistent with the previous findings in humans. Our findings may provide new insight into the relationship between *REG3G* and microbial colonization in the small intestine of newborn calves. There were no age-related changes in the expression of lineage-specific genes related to total T cell number in either jejunum or ileum during the 42 days’ post-partum. It is known, however, that there is an increased number of mucosal T cells in the small intestine of calves when comparing 2–3 weeks’ old calves and 6 months’ old calves [10]. It was surprising to observe a significant increase in the expression of gene (*FOXP3*) related to Tregs from D0 to D7 in both the jejunum and ileum. There is evidence that Tregs play a significant role in suppressing immune responses to commensal bacteria [30]. The number of Tregs in the colon increased following bacterial colonization in mice [31] and circulating Tregs increase rapidly in newborn human infants during the first days of life [32]. The probable increase in Tregs in both the ileum and jejunum of newborn calves during the first week of life suggests a possible adaptive response to maintain gut immune homeostasis following microbial colonization.

In addition to noted changes in the adaptive immune response, *IL8* and *IL10* expression were also up-regulated within the first week. IL10 is a well-studied anti-inflammatory cytokine [33] and the colonization of commensal bacteria, such as *Bifidobacterium sp*., stimulates the increased expression of *IL10* [34]. The upregulation of *IL10* within the first week would be consistent with repression the host inflammatory response and the induction of Tregs during microbial colonization. In contrast, IL8, a pro-inflammatory cytokine, recruits neutrophils from intravascular to interstitial sites and directly activate neutrophils [35]. The co-upregulation of *IL8* and *IL10* at one week postpartum indicates that the host may be reaching a balance in terms of the inflammatory response, which is crucial for immune homeostasis in the small intestine. Similarly, the expression of genes encoding PRRs, such as *TLR6* and *NLRP3* increased significantly in both jejunum and ileum within the first week. TLR6 forms a heterodimer with TLR2, recognizing diacylated lipopeptide from bacterial cell walls [36] and TLR6 stimulation of DCs can induce Treg cell development [37]. The significant correlation between *TLR6* expression and total bacterial number (*TLR6* and jejunal content-associated total bacteria at D7, *r* = −0.85, *P* < 0.05; *TLR6* and jejunal tissue-associated total bacteria at D21 and D42, *r* = −0.94 and *r* = −0.87, *P* < 0.05) in the jejunum, would be consistent with TLR6’s role in regulating host responses to the dynamic changes in the microbiome population. In addition to the membrane-bound TLRs, increased expression of *NLRP3* was also observed in both jejunum and ileum within the first week post-partum. NLRP3 forms an inflammasome complex in the cytoplasm, which can be activated by commensal bacteria-produced adenosine triphosphate, leading to downstream host responses such as the differentiation of T helper 17 cells (Th17) [24]. Th17 can produce a subset of cytokines, such as IL17A, IL17F, IL22, and IL26, which are essential to the mucosal immune responses [38]. The upregulation of *NLRP3* within the first week indicates its potential roles in responding to the microbial colonization and modulating the host response in the small intestine of calves during the postnatal period. However, there is limited knowledge regarding the function of NLRs in the mucosal immune system of ruminants and further studies are needed to elucidate their role in host-microbial interactions in the intestine of neonatal calves.

As described above, we observed diverse alterations in gene expression that may be vital for mucosal immune system development in ruminants during the first week of life, some of which could be associated with commensal bacterial colonization. We also investigated whether miRNAs, the non-coding RNAs that regulate gene expression, could also be one of the mechanisms regulating early development of the mucosal immune system. Similar to host mRNAs, the most significant changes in miRNAs expression in the small intestine were also observed during the first week of life [12]. In this study, we further identified mRNA-miRNA pairs which might be associated with microbial colonization and host immune functions. For example, the expression of miR-335 was down-regulated in the ileum during the first week post-partum and overexpression of miR-335 can repress the expression of *CLDN1*, *CLDN4* and *OCLN* [39]. Indeed, the increased expression of *CLDN1*, *CLDN4* and *OCLN* suggested that the downregulation of miR-335 together with dynamic changes in the gut microbiota may play a role in regulating the expression of TJ proteins during the first week of life. Furthermore, miR-335 has also been shown to repress the expression of *IL8* [39]. Therefore, the co-incidental upregulation of *IL8* at this time would be consistent with the downregulation of miR-335 within the first week post-partum. Another potential pairing identified was the downregulation of miR-100, while its predicted target, *NLRP3* [40], was upregulated during the first week of life. Collectively, this analysis suggests that miRNAs may be one mechanism by which host-microbial interactions regulate expression of genes involved in gut permeability, cytokine expression, and pathogen sensing during early life. However, further target validation experiments using in vitro or in vivo models will provide more conclusive understanding on the regulatory roles of these miRNAs in predicted target genes.

## Conclusion

This is the first study to analyze the whole transcriptome in the jejunal and ileal regions of the small intestine of calves during the early post-natal period. We observed regional expression difference in immune-related genes between the jejunum and ileum in the neonatal mucosal immune system and differences in the expression of mucosal immune-related genes. Furthermore, we provide evidence that some of these transcriptional changes are significantly associated with microbial colonization and miRNAs may play a role in regulating these events. We report that JE-enriched immune function related-genes were related primarily to the complement functional pathway, the expression of TJ protein genes and IgA complex genes was higher in the jejunum. In contrast, the expression of TLR and NOD-like receptor genes was lower in the jejunum than in the ileum. These regional transcriptome differences support the conclusion that the jejunum but not the ileum plays an important role in the adaptive mucosal immune system. These regional differences in early immune function may provide a potential explanation for why enteric infections occur primarily in the ileal region of the newborn calf. Furthermore, our transcriptomic analysis reveals that the first week post-partum is a very dynamic developmental period for both the intestinal epithelial barrier and the mucosal immune system. The expression of genes related to tight junction proteins, antimicrobial peptides, NOD-like receptors, regulatory T cells, and cytokines undergo dynamic changes when comparing 7-day-old to newborn calves. Furthermore, there were strong correlations between mucosal immune-related genes and the total bacterial population in different gut regions at different ages. This evidence implicates microbial colonization as one important factor modulating this early host gene expression. Moreover, we report that miRNAs, such as miR-335 and miR-100, may be one of the mechanisms involved in regulating this early mucosal immune system development in the small intestine of neonatal calves. Collectively, these data provide a baseline to begin investigating whether significant deviation from these early developmental effects have either short- or long-term effects on mucosal immune function and host-microbiome interactions.

## Methods

### Animals and tissue sample collection

Jejunum and ileal tissue samples were collected from Holstein bull calves at 30 min after birth (D0; *n* = 3), 7 days post-partum (D7; *n* = 5), 21 days post-partum (D21; *n* = 5) and 42 days post-partum (D42; *n* = 5) (Dairy Research and Technology Center, University of Alberta). All experimental protocols were reviewed and approved by the Livestock Animal Care Committee of the University of Alberta (Protocol No. AUP00001012) and all procedures were conducted following the guidelines of the Canadian Council on Animal Care. All samples were collected within 30 min after calves were euthanized using captive bolt gun. D0 samples were collected from newborn calves within 30 min after delivery and without ingestion of colostrum. All the other calves were fed 2 L of colostrum within 2 h after birth, and received another 2 L of colostrum at 12 h after birth. The D7 calves were fed 4 L milk/day and D21 and D42 calves were fed 4 L milk/day with ad libitum access to calf starter (23 % CP and 4 % ether extract with a guaranteed minimum 19.5 % NDF, 27.1 % starch; Wetaskiwin Co-Op Country Junction, Wetaskiwin, AB, Canada) from D14. The jejunum was defined as 100-cm distal to the pyloric sphincter and a 10-cm segment of intestine was collected. The ileum was defined as the 30-cm segment proximal to the ileo-cecal fold and a 10-cm segment of tissue was collected. All tissue samples, with the exception of D0 samples, were rinsed three times with sterile phosphate buffered saline (PBS, pH = 7.0) buffer to remove ingesta. Tissues were snap-frozen in liquid nitrogen and stored in−80 °C.

### RNA isolation

Tissue samples were ground into powder while immersed in liquid nitrogen in a frozen mortar prior to RNA extraction. Total RNA was extracted from 80 mg of tissue powder using mirVana™ miRNA Isolation Kit (Ambion, Carlsbad, CA) following the manufacturer’s instructions. The quality and quantity of the RNA were determined using the Agilent 2100 Bioanalyzer (Agilent Technologies, Santa Clara, CA) and Qubit 2.0 Fluorometer (Invitrogen, Carlsbad, CA), respectively. RNA samples with an integrity number (RIN) greater than 7.0 were used for library construction.

### RNA-Seq library construction and sequencing

Total RNA (1.0 μg) from each sample was used to construct RNA-Seq libraries with a unique index using the TruSeq mRNA Sample Preparation kit (Illumina, San Diego, CA) according to the manufacturer’s instruction. Qubit 2.0 Fluorometer (Invitrogen, Carlsbad, CA) was performed for library quantification. cDNA libraries were sequenced at Génome Québec (Montréal, Canada) using the Illumina HiSeq 2000 system (Illumina). Sequencing was performed as 100 bp paired-end reads. All reads were demultiplexed according to their index sequences with CASAVA version 1.8 (Illumina), and reads that did not pass the Illumina chastity filter were discarded.

### RNA-Seq reads mapping and annotation

RNA-Seq reads were aligned to the bovine genome (UMD 3.1) using Tophat 2.0.10 with default parameters [41, 42]. The number of reads mapped to each gene was counted by htseq-count (http://www-huber.embl.de/users/anders/HTSeq/) based on the annotation from ENSEMBL (http://uswest.ensembl.org/) bovine gene annotation v75.30. The expression level of mRNAs in each sample were calculated by normalizing reads number to counts per million reads (CPM) by the following formula: CPM = (gene reads number/total mapped reads number per library) × 1,000,000.

### Identification of differentially expressed genes

Identification of DE genes was performed by edgeR [13]. Regionally DE genes were identified by comparing mRNA expression of the jejunum with the mRNA expression of ileum at each time point (D0, D7, D21, and D42). Temporally DE genes were identified by comparing any two adjacent developmental stages (D7 *vs* D0; D21 *vs* D7; D42 *vs* D21). The significantly DE mRNAs were declared at fold change ≥ 2 and false discovery rate (FDR) < 0.05, and the FDR was calculated based on Benjamini and Hochberg multiple testing correction [43].

### Analysis of specific genes related to immune system and barrier function

The immune-related genes list was obtained from ImmPort database [44]. Genes encoding the subunits of cluster of differentiation 3 (*CD3D*, *CD3E* and *CD3G*) and *CD247* were selected as lineage-specific markers for T cells [45, 46], while genes encoding *CD79A* and *CD79B* were selected as lineage markers for B cells [47]. The gene encoding forkhead box P3 (*FOXP3*) was selected as a lineage-specific marker for regulatory T cells [48]. Genes that belonged to tight junction (TJ) protein families were selected for further analysis, such as the claudin (CLDN) family, zonula occluden (ZO) family, and junctional adhesion molecule (JAM) family. Genes encoding β-defensin (*DEFB*) and regenerating islet-derived 3 γ (*REG3G*) were selected to analyze antimicrobial peptide (AMP) gene expression patterns [28]. The genes encoding toll-like receptors (TLRs) and NOD-like receptors (NLRs) were selected to analyze the innate immune sensing-related genes. The cytokine list was obtained from Kyoto Encyclopedia of Genes and Genomes (KEGG) pathway annotation “Cytokine-cytokine receptor interaction”.

### Quantification of IgA concentration in the small intestinal lumen

IgA concentration was measured using Bovine IgA ELISA Quantitation Set (Bethyl Laboratories, Montgomery, USA) following the manufacturer’s instructions. The intestinal content samples were centrifuged and the supernatants were collected for further measurements. A reference standard of bovine serum, containing 0.11 mg/ml IgA was used to calibrate the assay for the samples. The absorbance was measured with the SpectraMax M3 system (Molecular Devices, Sunnyvale, USA).

### Functional analysis

The Gene Ontology (GO) terms and KEGG pathways enrichment was performed using Database for Annotation, Visualization and Integrated Discovery (DAVID, http://david.abcc.ncifcrf.gov) [49]. Each analysis was performed using the functional annotation clustering option, and significant GO terms and KEGG pathways were selected at *FDR* < 0.05 and molecules number > 5.

### Integrated analysis of miRNA and mRNA expression

All the results for miRNAs were obtained from a previous study which utilized the same samples to profile miRNA expression [12]. The DE miRNAs and DE mRNAs in D7 *vs* D0 were used in this study due to the greater number of DE miRNAs and mRNAs than any other comparison group. The regulatory relationship between DE miRNAs and DE mRNAs was identified based on two criteria: computational target prediction and the DE miRNAs that had opposite expression patterns from their predicted targets. Target genes of miRNAs were predicted by TargetScan Release 6.0 (default parameters, http://www.targetscan.org/) and miRanda (total score > 145, total energy < −10 kcal/mol, http://www.microrna.org/). Furthermore, the miRTarBase was used to identify the experimentally validated miRNA-mRNA regulatory pairs [14].

### Experimental validation of mRNA expression by RT-qPCR

A total of 12 genes were selected to validate their regionally and temporally DE patterns. *CLDN1*, *CLDN4* and *OCLN* from TJ proteins; *TLR2*, *TLR4*, *TLR6* and *TLR10* from the TLR gene family; *FOXP3* as the Treg marker; *IL8*, *IL10*, *TGFB* and *TNFA* from cytokines; were selected for RT-qPCR expression analysis. All the primer information was summarized in supplementary files (Additional file 10). All protocols were performed as described in a previous study [11]. The RT-qPCR was performed using SYBR Green (Fast SYBR® Green Master Mix; Applied Biosystems) to detect mRNA relative expression. Fluorescence signal was detected with the StepOnePlus™ Real-Time PCR System (Applied Biosystems). Relative gene expression (ΔCq value) was calculated based on quantification cycle (Cq) of reference gene (β-actin) and target gene (ΔCq = Cq _target gene_ − Cq _reference gene_).

### Correlation analysis between host gene expression and total bacterial number

The ΔCq values obtained from RT-qPCR results and the 16S rRNA gene copy numbers of total bacteria in the small intestinal content and tissue obtained from our previous study [12] were subjected to correlation analysis. Pearson’s correlations were performed between host gene expression (12 genes with RT-qPCR results) and total bacterial number in the content as well as between host gene expression and total bacterial number in the tissue at different ages. R software (version3.0) was used to calculate the correlation coefficients (r) and *P* values, and significant correlations were declared at r > 0.8 or < −0.8; *P* < 0.05.

### Statistical analysis

The significantly regionally and temporally DE genes (RNA-Seq results) were declared at fold change > 2 and FDR < 0.05, and the FDR was calculated based on Benjamini and Hochberg multiple testing correction [41]. The RT-qPCR results were analyzed using ANOVA, and significances were declared at *P* < 0.05. R software (version3.0) was used to for correlation analysis, and significant correlations were declared at r > 0.8 or < −0.8; *P* < 0.05.

### Data Submission

All the RNA-Seq data were deposited in the publicly available NCBI’s Gene Expression Omnibus Database (http://www.ncbi.nlm.nih.gov/geo/). The data are accessible through GEO Series accession number GSE74329 (http://www.ncbi.nlm.nih.gov/geo/query/acc.cgi?acc=GSE74329).

## Abbreviations

AMP, antimicrobial peptide protein; CATHL, Bos taurus cathelicidin; CD, Cluster of Differentiation; CLDN, claudin; CPM, counts per million mapped reads; DE genes, differentially expressed genes; DEFB, β-defensin; FC, fold change; FDR, false discovery rate; FOXP3, forkhead box P3; GO, Gene Ontology; IGJ, immunoglobulin J chain; IL, interleukin; IL, the ileum; JAM, junctional adhesion molecule; JE, the jejunum; KEGG, Kyoto Encyclopedia of Genes and Genomes; miRNAs, microRNAs; NLR, NOD-like receptor; PCA, principal component analysis; PIGR, polymeric immunoglobulin receptor; PPs, Peyer’s patches; REG3G, regenerating islet-derived 3-γ; SIGIRR, single Ig IL-1-related receptor; TGFB, transforming growth factor β; Th17, T helper 17 cells; TJ, tight junction; TLRs, Toll-like receptors; TNFA, tumor necrosis factor α; TOLLIP, toll interacting protein; Tregs, regulatory T cells; ZO, zonula occluden

## Additional files

Additional file 1: DAVID function of commonly expressed genes in the jejunum and ileum. (XLSX 15 kb) Additional file 2: Regionally DE genes list. (XLSX 282 kb) Additional file 3: Temporally DE genes list. Sheet 1. Temporally DE genes in the jejunum. Sheet 2. Temporally DE genes in the ileum. (XLSX 186 kb) Additional file 4: GO terms enrichment for each temporally DE gene expression pattern. (PDF 404 kb) Additional file 5: All the immune-related gene list. (XLSX 1650 kb) Additional file 6: The KEGG pathway of regional DE immune-related genes. (XLSX 34 kb) Additional file 7: The temporally DE analysis for the immune-related genes. Sheet 1. Temporally DE immune-related genes list in the jejunum. Sheet 2. KEGG pathway analysis of Temporally DE immune-related genes in the jejunum. Sheet 3. Temporally DE immune-related genes list in the ileum. Sheet 4. KEGG pathway analysis of Temporally DE immune-related genes in the ileum. (XLSX 45 kb) Additional file 8: DE miRNA information used for this study. Sheet 1. DE miRNAs in the jejunum. Sheet 2. DE miRNAs in the ileum. (XLSX 13 kb) Additional file 9: Experimentally validated miRNA-mRNA regulatory pairs. Sheet 1. miRNA-mRNA regulatory pairs in the jejunum. Sheet 2. miRNA-mRNA regulatory pairs in the ileum. (XLSX 10 kb) Additional file 10: Primer information for RT-qPCR validation. (XLSX 9 kb)
