# Supplementary material for: Transcriptome analysis reveals regional and temporal differences in mucosal immune system development in the small intestine of neonatal calves
Source: BMC Genomics. 2016 Aug 11;17:602. doi: 10.1186/s12864-016-2957-y (PMC4981982; doi:10.1186/s12864-016-2957-y)
Supplement: Additional file 4: — GO terms enrichment for each temporally DE gene expression pattern. (PDF 404 kb) [file 12864_2016_2957_MOESM4_ESM.pdf]

JE	IL		JE	IL
0.05	0.00	DDD	**Transport**	**NA**
0.12	0.13	DDN	**Nitrogen compound metabolic process**	**Cholesterol homeostasis**
0.00	0.16	DDU	**NA**	**Tissue development**
0.27	0.00	DND	**Transport**	**NA**
7.58	6.51	DNN	**Developmental process**	**Developmental process**
0.05	0.78	DNU	**Phosphorylation**	**Metabolic process**
0.02	0.01	DUD	**Transport**	**NA**
0.08	0.10	DUN	**Proteolysis**	**Translation**
0.01	0.01	DUU	**Carbon dioxide enzyme**	**Chemokine**
0.02	0.01	NDD	**Protein-lipid complex remodeling**	**Epithelial cell development**
0.47	0.51	NDN	**Transport**	**Transport**
0.01	0.49	NDU	**Structural molecule**	**Cell adhesion**
0.57	0.15	NND	**Metabolic process**	**Immune system process**
80.66	86.24	NNN	**Metabolic process**	**Metabolic process**
0.65	1.48	NNU	**Cellular morphogenesis**	**Muscle contraction**
0.03	0.03	NUD	**Lipid transport**	**Defense response**
0.34	0.35	NUN	**Immune system process**	**Defense response**
0.04	0.01	NUU	**Response to bacterium**	**Structural molecule**
0.01	0.00	UDD	**Transport**	**NA**
0.21	0.26	UDN	**Immune response**	**NA**
0.02	0.07	UDU	**Cell fraction**	**Cell development**
0.40	0.01	UND	**Immune response**	**NA**
7.73	2.58	UNN	**Immune system process**	**Immune system process**
0.18	0.12	UNU	**Immune system process**	**Cytokine production**
0.09	0.01	UUD	**Intrinsic to membrane**	**Cell death**
0.35	0.01	UUN	**Immune response**	**Transcription factor**
0.06	0.00	UUU	**Intrinsic to membrane**	**NA**
